# A Histological Comparison Between Anterior Cruciate Ligament Remnant Tissue, Anatomically Reconstructed Graft, and Non-Anatomically Reconstructed Graft

**DOI:** 10.7759/cureus.13016

**Published:** 2021-01-30

**Authors:** Tsuneari Takahashi, Masashi Kimura, Hiroshi Higuchi, Kosuke Suzuki, Yuichiro Yamada, Katsushi Takeshita

**Affiliations:** 1 Orthopaedic Surgery, Jichi Medical University, Shimotsuke, JPN; 2 Orthopaedic Surgery, Zenshukai Hospital, Maebashi, JPN; 3 Orthopaedic Surgery, Asakura Sports Rehabilitation Clinic, Maebashi, JPN

**Keywords:** anterior cruciate ligament reconstruction, arthroscopy, histological evaluation, remnant tissue, revision surgery

## Abstract

Introduction: To our knowledge, no studies have investigated the histological comparison between primary injured anterior cruciate ligament (ACL), initially anatomically reconstructed grafts and non-anatomically reconstructed grafts at the time of revision ACL reconstruction. The purpose of this study was to histologically clarify the differences between ACL remnant tissue, reconstructed graft after anatomic double-bundle ACL reconstruction, and reconstructed graft after non-anatomic single-bundle ACL reconstruction.

Methods: This histological study included five patients after anatomic double-bundle ACL reconstruction, three patients after non-anatomic single-bundle ACL reconstruction performed who injured their operated knees again, and five patients who injured their ACL for the first time and agreed to participate. All of the grafts and ACL remnant tissue were harvested, stained with hematoxylin and eosin, S-100, and alpha smooth muscle actin and evaluated using light microscopy.

Results: There was no area of necrosis in the reconstructed graft after an anatomic double-bundle ACL reconstruction. However, there were obvious areas of necrosis in the reconstructed graft after non-anatomic single-bundle ACL reconstruction. Additionally, the collagen fibers were more longitudinally oriented, and most cells were spindle shaped like those in ACL remnant tissue after an anatomic double-bundle ACL reconstruction in contrast with the finding of the grafts after non-anatomic single-bundle ACL reconstruction.

Conclusion: Initially reconstructed graft after an anatomic double-bundle ACL reconstruction may be beneficial if preserved at the time of the revision surgery.

## Introduction

Anterior cruciate ligament (ACL) reconstruction using the hamstring tendon or the central third of the bone-patellar tendon-bone (BTB) autograft has been widely performed. However, patient satisfaction after ACL reconstruction has not been high because of postoperative laxity of the knee [1]. One plausible reason for this postoperative laxity is persistent necrosis of the graft. Delay et al. reported an autopsy after ACL reconstruction using BTB autograft after 18 months. There were persistent areas of necrosis and acellularity deep within the substance of the graft [2]. Weiss et al. described that myofibroblasts might be involved in crimp formation and be an integral part of normal ligament tissue. Furthermore, the shape of myofibroblasts may further indicate the contractile potency of the extracellular matrix, thus presenting a dynamic and variable crimp [3]. Therefore, persistent areas of necrosis and acellularity within the graft may indicate insufficient remodeling and recovery of a functionally normal ACL. In the early last decade, anatomical and biomechanical studies have been conducted, and anatomical ACL reconstruction, such as anatomical single- or double-bundle ACL reconstruction, was developed and clinical results have been reported to be superior to conventional ACL reconstruction [4], but important issues, such as re-injury and contralateral ACL injury, remain to be solved [5].

To date, ACL remnant tissue has attracted attention. Sonnery-Cottet et al. described that ACL remnants contained well-vascularized synovial sheet, numerous fibroblasts and myofibroblasts, and mechanoreceptors in their histological study [6]. Remnant tissue preservation at the time of ACL reconstruction was reported beneficial to accelerate the remodeling phase, re-innervation, and revascularization of the graft [7]. However, it still remains uncertain whether initially reconstructed grafts should be preserved at the time of revision ACL reconstruction. If there are enough myofibroblasts, nerve organs, or vessels in the graft or surrounding synovium like ACL remnant tissue, even though the graft is stretched and injured, the preservation of the initial graft may be beneficial as well as remnant preservation at the time of the revision ACL reconstruction.

Therefore, we decided to perform a histological analysis to compare ACL remnant tissue, reconstructed grafts after anatomic double-bundle ACL reconstruction, and reconstructed grafts after non-anatomic single-bundle ACL reconstruction. The purpose of this study was to histologically clarify the differences between ACL remnant tissue, reconstructed graft after anatomic double-bundle ACL reconstruction, and reconstructed graft after non-anatomic single-bundle ACL reconstruction.

## Materials and methods

Patient selection

This case series study was conducted in the Department of Orthopaedic Surgery of Gunma Sports Medicine Research Center, Zenshukai Hospital (Maebashi, Japan). Five patients, after anatomic double-bundle ACL reconstruction performed at our institution, who injured their operated knees again (Group A-DB); three patients, after non-anatomic single-bundle ACL reconstruction performed at another institution, who injured their operated knees again (Group NA-SB); and five patients, who injured ACL for the first time from April 2016 to Match 2017 and agreed to participate in this histological study (Group R), were included. Five patients after anatomic double-bundle ACL reconstruction were followed up for one year or more and successfully returned to their preoperative activity. These patients had been diagnosed by using clinical parameters, such as a positive Lachman test, Telos® stress radiography (Telos GmbH®, Laubscher, Holstein, Switzerland), and magnetic resonance imaging findings. The physical examination and radiological interpretation were performed by experienced orthopedic surgeons who were not otherwise involved in this study.

Definition of anatomic double-bundle ACL reconstruction

Patients of re-injury after anatomic double-bundle ACL reconstruction underwent CT before revision surgery to evaluate whether the femoral and tibial bone tunnels were anatomically located [8,9].

Definition of non-anatomic single-bundle ACL reconstruction

Patients of re-injury after non-anatomic single bundle ACL reconstruction without remnant tissue preservation underwent CT before revision surgery to evaluate the location of the bone tunnels. All patients had a femoral bone tunnel located at noon position thus being diagnosed as ‘non-anatomic’ procedures.

Background comparisons between Group A-DB and Group NA-SB

Age, gender, reason for re-injury (contact/non-contact), graft source at the time of primary ACL reconstruction (hamstring/BTB), time from initial reconstruction to histological evaluation, and time from re-injury to revision surgery were compared between the groups. Mann-Whitney U-test and Fisher’s exact test were used for comparison and significant level was set at P<0.05.

Histological and immunohistochemical evaluations

All of the injured graft in Group A-DB and Group NA-SB, and ACL remnant tissue in Group R exhibited weak tension when probed during routine arthroscopic evaluation. The entire injured graft in Group A-DB and Group NA-SB and ACL remnant tissue in Group R were harvested using a sharp scalpel. Care was taken not to injure the ACL tibial enthesis-lateral meniscus complex [10]. In addition, AM grafts in the tibial bone tunnel and surrounding cancellous bone in Group A-DB and Group NA-SB were harvested using round chisel to evaluate the interface tissue between the graft and cancellous bone. The retrieved specimens were fixed by using a 10% neutral buffered formalin solution for 24 hours at 4°C. Then, 5-mm-thick longitudinal sections were cut in the sagittal plane along the longest axis of the graft and stained with hematoxylin and eosin for histomorphological observation. Mechanoreceptors and nerve endings were evaluated using S100 protein staining according to the criteria of Freeman [11,12]. Alpha smooth muscle actin (SMA) staining was used to identify blood vessels. The sections were evaluated using light microscopy (BioRevo BZ-9000; Keyence Corp., Itasca, IL) [7].

## Results

There were no significant differences concerning background between the groups (Table 1).

**Table 1:** Patient characteristics A-DB, anatomic double-bundle; NA-SB, non-anatomic single-bundle; BTB, bone-patellar tendon-bone *Data are expressed as mean (standard deviation).

Parameters	A-DB (n=5)	NA-SB (n=3)	P value
Age*	39.2 (13.4)	28.0 (13.9)	0.653
Sex (male/female)	2/3	2/1	1.0
Reason for re-injury (contact/non-contact)	0/5	1/2	0.375
Initial graft source (hamstring/BTB)	5/0	1/2	0.375
Time from initial reconstruction to histological evaluation (weeks)*	627 (810)	431 (497)	1.0
Time from re-injury to surgery (days)*	98.3 (82.0)	87.7 (62.4)	0.706

Histological observations of the ACL remnant tissue

**Figure 1:**
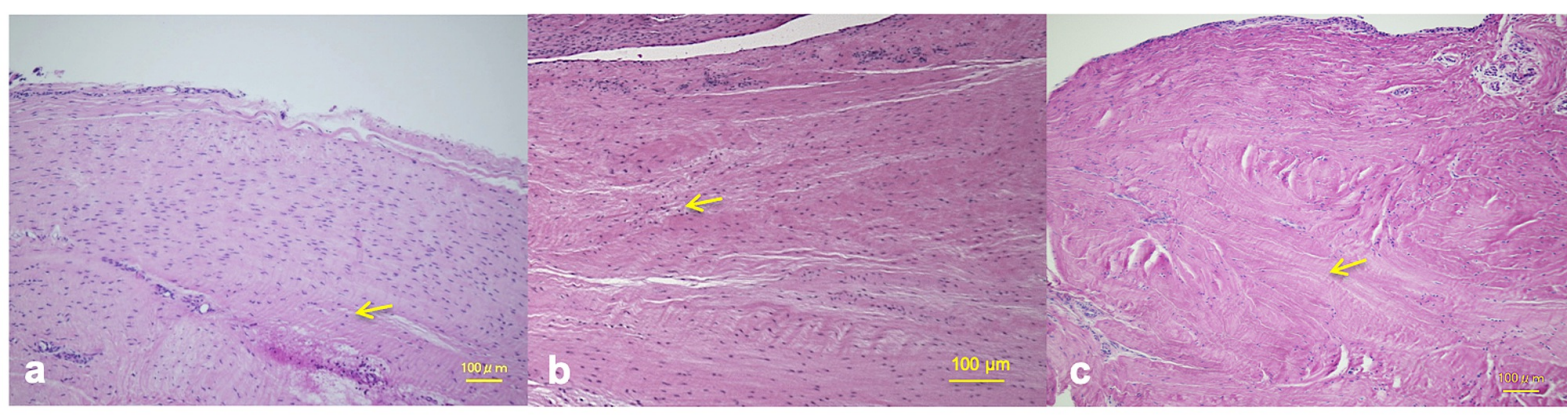
Histological observation of the superficial and core portions of the ACL remnant tissue and hamstring tendon autograft (a) The ACL remnant tissue was covered by thin synovial tissue. The collagen fibers were more longitudinally oriented, and many spindle-shaped cells, such as myofibrocytes, were scattered in the both the superficial and core portions of the ACL remnant tissue. Some cells in the core portion were spherical rather than spindle shaped. The yellow arrow indicates the spherically shaped cells. There were no areas of necrosis. (b) The graft after anatomic double-bundle ACL reconstruction was covered by thin synovial tissue. The collagen fibers were longitudinally oriented, and many spindle-shaped cells, such as myofibrocytes, were scattered in both the superficial and core portions of the graft. Some spherical cells were also scattered in both the superficial and core portions of the graft. The yellow arrow indicates the spherically shaped cells. There was a little acellular area in the core portion and there were no areas of necrosis. (c) The graft after non-anatomic single-bundle ACL reconstruction was covered by thin synovial tissue. The collagen fibers were irregularly rather than longitudinally oriented, and some spindle-shaped cells, such as myofibrocytes, and spherically shaped cells were scattered in the superficial portion of the graft. There was an area of obvious necrosis in the core portion of the graft. The yellow arrow indicates the area of necrosis. ACL, anterior cruciate ligament. Magnification X100, scale bar = 100 µm.

The ACL remnant tissue was covered by thin synovial tissue. The collagen fibers were more longitudinally oriented, and many spindle-shaped cells were scattered in both the superficial and core portions of the graft. Some were not spindle shaped but rather spherically shaped, and there was a little acellular area in the core portion (Figure 1a).

Blood Vessels in the ACL Remnant Tissue

**Figure 2:**
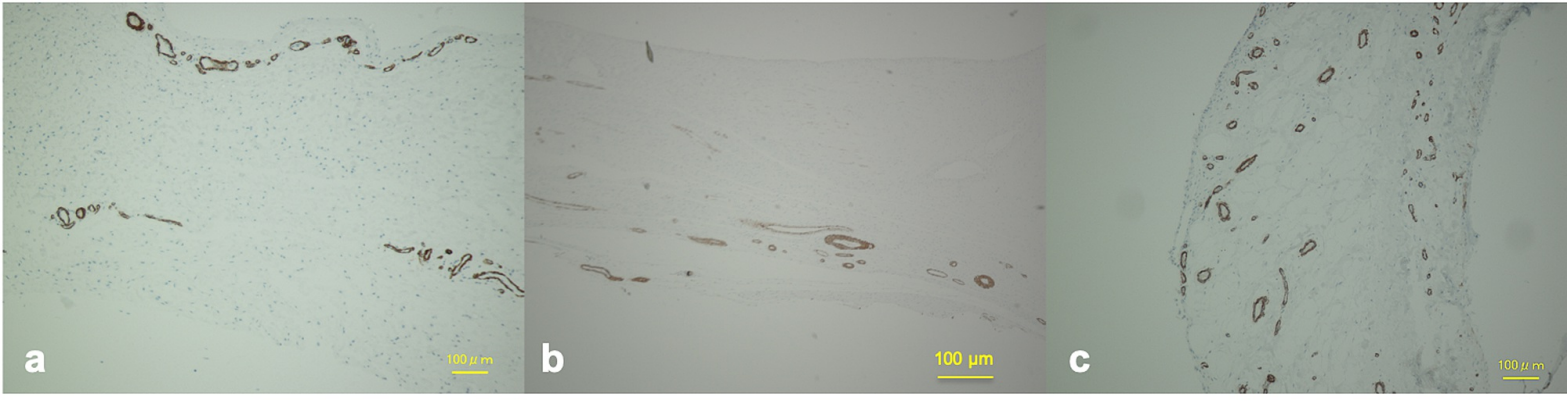
Immunohistochemical observation of the alpha SMA-positive blood vessel in the ACL remnant tissue and hamstring tendon autograft There were many alpha SMA-positive blood vessels observed in the ACL remnant tissue, reconstructed graft, and surrounding synovium: (a) ACL remnant tissue, (b) graft after anatomic double-bundle ACL reconstruction, (c) after non-anatomic single-bundle ACL reconstruction. ACL, anterior cruciate ligament; SMA, smooth muscle actin. Magnification X100, scale bar = 100 µm.

Many alpha SMA-positive blood vessels were observed in the ACL remnant tissue and surrounding synovium (Figure 2a).

Proprioceptive Organs in the ACL Remnant Tissue

**Figure 3:**
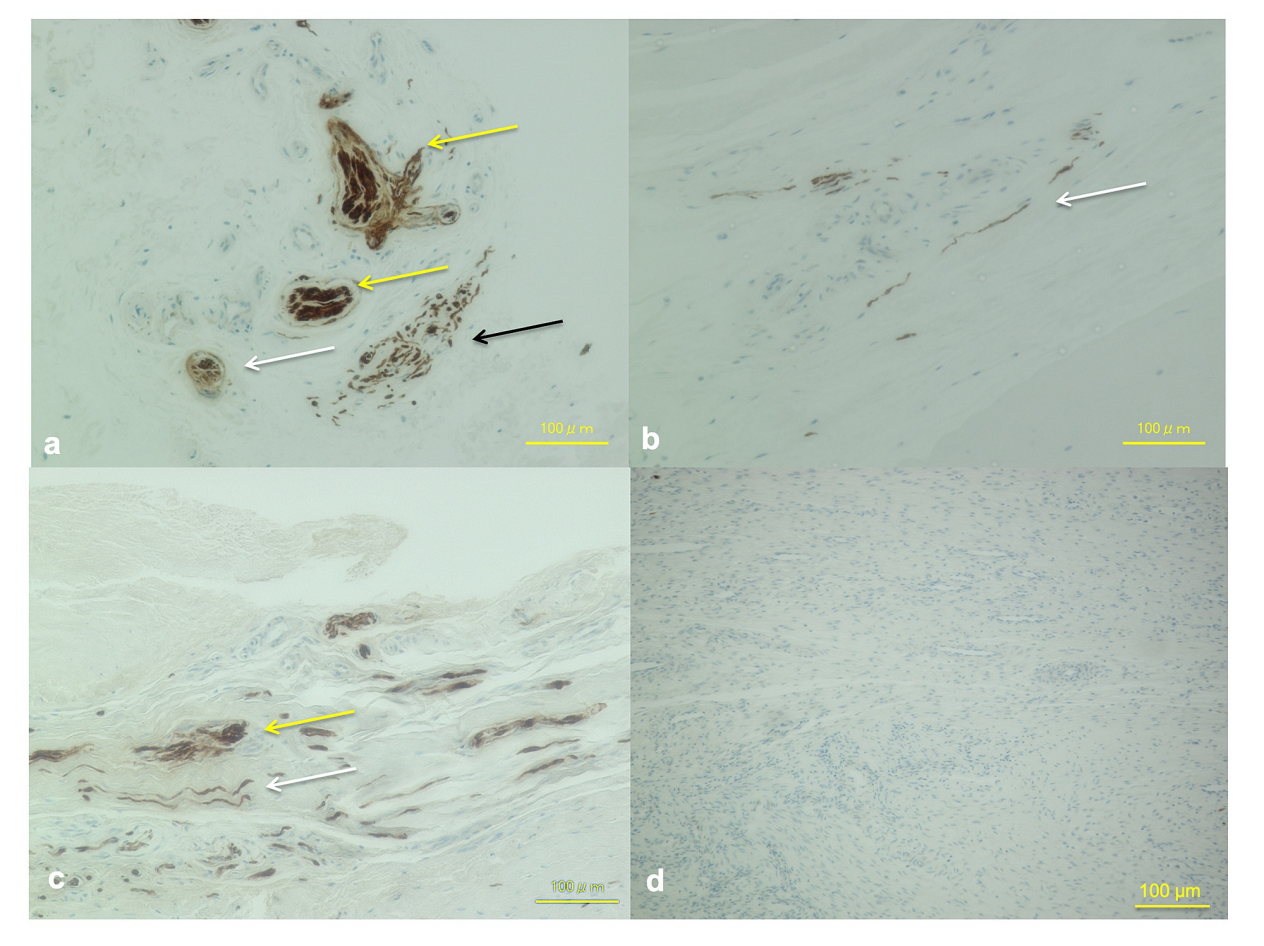
Immunohistochemical observation of the S100-positive mechanoreceptors in the ACL remnant tissue and hamstring tendon autograft (a) There were several mechanoreceptors found in the superficial area of the ACL remnant tissue and surrounding synovium: Type 1 mechanoreceptors (Pacini corpuscles, yellow arrows), Type 2 mechanoreceptors (Ruffini corpuscles, white arrow), and Type 3 mechanoreceptors (Golgi tendon organ, black arrow). (b) There were several Type 4 nerve organs (free nerve ending, white arrow) found in the mid-substance of the ACL remnant tissue. (c) There were several Type 4 nerve organs (free nerve ending, white arrow) found in the mid-substance after non-anatomic single-bundle ACL reconstruction. There were S100-positive corpuscles, but these were atypical according to the Freeman criteria. The yellow arrow indicates the atypical corpuscles. (d) There were no mechanoreceptors and free nerve endings in the graft after anatomic double-bundle ACL reconstruction. ACL, anterior cruciate ligament. Magnification X100, scale bar = 100 µm.

Type 1 mechanoreceptors (Pacini corpuscles) were found in 1 (20%) of five cases, Type 2 mechanoreceptors (Ruffini corpuscles) found in 2 (40%) of five cases, and Type 3 mechanoreceptors (Golgi tendon organ) were found in 1 (20%) of five cases (Figure 3a). These mechanoreceptors were found in the superficial area of the ACL and surrounding synovium; additionally, Type 4 nerve organs (free nerve ending) were found in the mid-substance of the ACL in 3 (60%) of five cases (Figure 3b).

Histological observations of the reconstructed ACL after anatomic double-bundle ACL reconstruction

**Figure 4:**
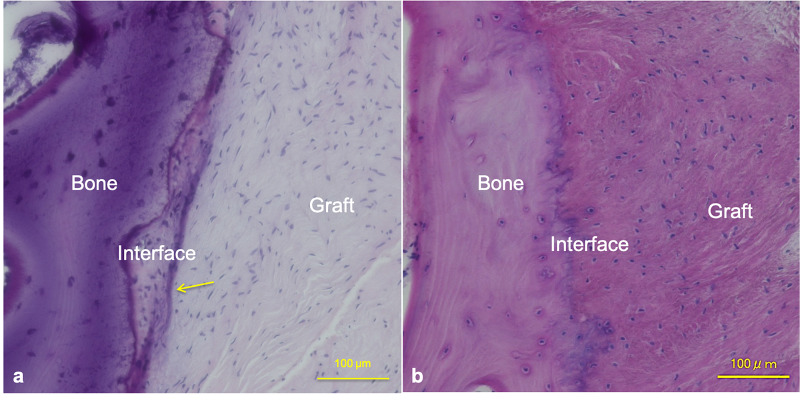
Histological observation of the bone-tendon autograft interface of the tibial bone tunnel (a) The collagen fibers of the graft in the tibial bone tunnel after anatomic double-bundle ACL reconstruction were more longitudinally oriented, and many spindle-shaped cells, such as myofibrocytes, were scattered. Biological fixation of the tibial bone tunnel between the graft was observed and showed collagen fiber continuities resembling Sharpey fibers and calcification around the graft. The yellow arrow indicates calcification between the graft and interface tissue. (b) The collagen fibers of the graft in the tibial bone tunnel after non-anatomic single-bundle ACL reconstruction were irregularly oriented and many spherically shaped cells were scattered. Biological fixation of the tibial bone tunnel between the graft was observed and showed collagen fiber continuities resembling Sharpey fibers; however, calcification between the graft and interface tissue was not observed. ACL, anterior cruciate ligament. Magnification X100, scale bar = 100 μm.

The graft was covered by thin synovial tissue. The collagen fibers were more longitudinally oriented, and many spindle-shaped cells were scattered in the superficial portion of the graft. In the core portion of the graft, some were spherically shaped rather than spindle shaped, and there was a little acellular area and no obvious area of necrosis (Figure 1b). In the tibial bone tunnel, biological fixation of the tibial bone tunnel between the graft was observed and showed collagen fiber continuities resembling Sharpey fibers and calcification around the graft (Figure 4a).

Blood Vessels in the Reconstructed Double-Bundle ACL

Many alpha SMA-positive blood vessels were observed in the reconstructed ACL and surrounding synovium (Figure 2b).

Proprioceptive Organs in the Reconstructed Double-Bundle ACL

No mechanoreceptors were found in the reconstructed ACL tissue and surrounding synovial tissue (Figure 3d).

Histological observations of the reconstructed ACL after non-anatomic single-bundle ACL reconstruction

The graft after non-anatomic single-bundle ACL reconstruction was covered by thin synovial tissue. The collagen fibers were irregularly rather than longitudinally oriented, and some spindle-shaped cells, such as myofibrocytes, and spherically shaped cells were scattered in the superficial portion of the graft. There was an area of obvious necrosis in the core portion of the graft (Figure 1c).

The collagen fibers of the graft in the tibial bone tunnel after non-anatomic single-bundle ACL reconstruction were irregularly oriented, and many spherically shaped cells were scattered. Biological fixation of the tibial bone tunnel between the graft was observed, as shown by collagen fiber continuities resembling Sharpey fibers; however, calcification between the graft and interface tissue was not observed (Figure 4b).

Blood Vessels in the Reconstructed Non-Anatomic ACL

Many alpha SMA-positive blood vessels were observed in the reconstructed ACL and surrounding synovium (Figure 2c).

Proprioceptive Organs in the Reconstructed Non-Anatomic ACL

There were several Type 4 nerve organs (free nerve ending, white arrow) found in the mid-substance after non-anatomic single-bundle ACL reconstruction in 1 (33%) of three cases. There were S100-positive corpuscles in 1 (33%) of three cases, but these were atypical according to the Freeman criteria (yellow arrow) (Figure 3c).

## Discussion

Stress shielding due to the ACL primary injury or re-injury after reconstruction affects the tendon matrix, specifically collagen molecules. It also affects fibroblasts, and subsequently, the proliferative fibroblasts significantly change the structure of collagen resulting in the deterioration of mechanical properties. These mechanisms may affect histological findings [13]. This histological study revealed that there were several similar histological findings between initially reconstructed graft after an anatomic double-bundle ACL reconstruction and primary injured ACL. There was no area of necrosis in the reconstructed graft using a hamstring tendon autograft after anatomic double-bundle ACL reconstruction as in the ACL remnant tissue. There were no significant differences between groups after anatomic double-bundle ACL reconstruction, and non-anatomic single-bundle ACL reconstruction concerning age, time from initial reconstruction to histological evaluation, and time from re-injury to surgery. On the other hand, there was an obvious area of necrosis in the reconstructed graft using a hamstring tendon autograft after a non-anatomic single-bundle ACL reconstruction. Additionally, the collagen fibers were more longitudinally oriented, and most cells were spindle shaped, like those in the ACL remnant tissue after an anatomic double-bundle ACL reconstruction. On the other hand, the collagen fibers were irregularly oriented, and some spindle-shaped cells were scattered only in the superficial portion of the graft after a non-anatomic single-bundle ACL reconstruction. There was also an area of obvious necrosis in the core portion of the graft that is not in accordance with past reports of single-bundle ACL reconstruction [2]. In past reports, the shape of myofibroblasts might have indicated the contractile potency of the extracellular matrix; thus, they present a dynamic and variable crimp [3]. So these findings may indicate better remodeling and result in better function of the reconstructed ACL after an anatomic double-bundle ACL reconstruction. Why there was no area of necrosis may partly be explained by the advantages of an anatomic double-bundle ACL reconstruction. On the other hand, results of immunohistochemical evaluations indicated that mechanoreceptors were not always regenerated even in anatomically reconstructed grafts and sometimes regenerated in a non-anatomically reconstructed graft. In a double-bundle ACL reconstruction, each graft was relatively thinner than that used in single-bundle ACL reconstruction. Kondo et al. reported a better outcome of anatomic double-bundle ACL reconstruction and speculated that the core portion of the graft in a double-bundle ACL reconstruction may be revascularized earlier than that of the graft in a single-bundle ACL reconstruction [14]. Therefore, there is a certain possibility that the anatomical bone tunnel and thinner graft contributed to better fiber orientation in Group A-DB.

We also clarified that the biological fixation of the tibial bone tunnel between the graft was observed and showed collagen fiber continuities resembling Sharpey fibers and calcification, as previously reported [15]. Micromotion of the hamstring graft inside the drilled canal may have a role in bone-tendon healing [16]. According to past reports, there might be an appropriate micromotion of the graft in the tibial bone tunnel, thus leading to biological fixation after an anatomic double-bundle ACL reconstruction. This is a plausible reason for better bone-tendon healing in Group A-DB.

This study had several limitations. First, not all of the patients who underwent revision or primary ACL reconstruction were included in this study because written informed consent was only obtained from patients for whom the first author (TT) could participate in the surgery. Second, the sample size of this study was small, so the authors did not perform power analysis for this study. In addition, patients after anatomic single-bundle ACL reconstruction were not included in this study because there were no patients who underwent revision ACL reconstruction after single-bundle ACL reconstruction during the study period. A comparison between anatomic single-bundle and non-anatomic single-bundle reconstructions is required, as anatomic single-bundle is now performed as the gold standard in the majority of countries. This constraint may have biased the results and will be our future interest to be clarified. Third, we could not resect the grafts from the bone outlet of tunnels nor detect any S100-positive mechanoreceptors in this study. Fromm et al. reported that revascularization was considerable by the 24th postoperative week and reinnervation was essentially complete by then in the graft [17]. Consequently, the results could possibly have been influenced because neurofilament-containing nerve fibers were preferentially located near the bony attachments of the ACL [18]; thus, the regeneration of nerve organs may follow the same course in the reconstructed graft. Fourth, to avoid injuring the ACL tibial enthesis-lateral meniscus complex, we could not evaluate the calcified cartilage area in the tibial insertion. The findings of this area are very important because ACL deficiency leads to chondrocyte apoptosis in that area [19]. So, if normal calcified cartilage area in the tibial insertion is observed, it may be beneficial to preserve the initial graft or at least the tibial portion because intact tibial insertion may cause the anterior drawer force to be applied gradually to the tibia relative to the femur [20]. In addition, this is also our future interest to see whether reconstructed graft fibers in patients after anatomic ACL reconstruction arise from the most posterior part of the 'duck-foot' in a flat and 'c-shaped' way as reported by Oka et al. [21].

Beyond these limitations, however, to our knowledge, this study is the first to evaluate reconstructed ACLs after an anatomic double-bundle ACL reconstruction using hamstring tendon autografts even though the specimens were obtained from the re-injured patients. Regarding the clinical relevance, a potential benefit of the initially reconstructed graft after an anatomic double-bundle ACL reconstruction is that it may accelerate remodeling and revascularization at the time of revision ACL reconstruction. Further research is needed to investigate the bioactivity of initial grafts.

## Conclusions

Initially reconstructed graft after an anatomic double-bundle ACL reconstruction showed different histological findings compared with non-anatomically reconstructed grafts at the time of the revision surgery. The graft of the anatomical group may be structurally superior to that of the non-anatomical group, but there are problems in nerve terminal regeneration.
